# Soil and Vegetation Drive Sesquiterpene Lactone Content and Profile in *Arnica montana* L. Flower Heads From Apuseni-Mountains, Romania

**DOI:** 10.3389/fpls.2022.813939

**Published:** 2022-01-28

**Authors:** Anja Greinwald, Martin Hartmann, Jörg Heilmann, Michael Heinrich, Rainer Luick, Albert Reif

**Affiliations:** ^1^Nature Conservation, University of Applied Forest Science, Rottenburg, Germany; ^2^Vegetation Science, University of Freiburg, Freiburg, Germany; ^3^Pharmaceutical Biology, University of Regensburg, Regensburg, Germany; ^4^Pharmacognosy and Phytotherapy, University College London (UCL) School of Pharmacy, London, United Kingdom

**Keywords:** *Arnica montana*, base saturation, lactones, nutrient availability, sesquiterpenes, soil pH, species richness, vegetation cover

## Abstract

*Arnica montana* L. (*AM*, Asteraceae) is a perennial, herbaceous vascular plant species of commercial importance. The flower heads’ pharmacological properties are attributed mainly to sesquiterpene lactones (SLs), with phenolic acids and flavonoids also considered of relevance. The botanical drug is still partly collected in different European mountain regions. The SL content can be influenced by genetic factors and environmental conditions (altitude, temperature and rainfall). Surprisingly, the influence of the soil on SL-content have rarely been investigated. However, the soil determines the occurrence, distribution and overall fitness of *AM*. Equally, environmental factors are crucial determinants for the biosynthesis and fluctuations in plant secondary metabolites. Therefore, different abiotic (pH, C/N ratio, base saturation, cation exchange capacity) and biotic (species richness, vegetation cover) parameters need to be assessed as potential drivers of the variable content of A*M’s* secondary metabolites. Consequently, we developed an *in situ* experimental design aiming to cover a wide range of soil pH conditions. We detected and investigated different *AM* populations growing in grassland on acidic soils, on siliceous as well as calcareous geologies within the same geographical region and altitudinal belt. The total SL content and most single SL contents of the *AM* flower heads differed significantly between the two geologies. *AM* flower heads of plants growing on loam on limestone showed a significant higher total SL content than the flower heads of plants growing in siliceous grasslands. Furthermore, the SL contents were significantly correlated with geobotanical species richness and vegetation cover pointing toward an effect of species interactions on the production of SLs. Moreover, the ratios of the main SLs helenalin to dihydrohelenalin esters were significantly correlated to environmental parameters indicating that SL composition might be a function of habitat conditions. The findings of this study shed light upon the often ignored, complex interactions between environmental conditions and plant secondary metabolites. We highlight the importance of both abiotic and biotic habitat parameters for SLs in *AM*.

## Introduction

*Arnica montana* L. (Asteraceae) is a perennial, herbaceous vascular plant species and characteristic of nutrient-poor acid meadows, pastures and heaths. It is endemic to Europe with predominant distribution in mountainous areas from South-Norway, Latvia in the Baltic region to South-Portugal, the northern Apennines in Italy and the southern Carpathians in Romania (Kahmen and Poschlod, 2000; Luijten et al., 2000; Falniowski et al., 2011; Maurice et al., 2012; Hollmann et al., 2020). For centuries *A. montana* has been a well-known, commercial medicinal plant used for various diseases such as arthritis, rheumatic disorders, inflammation and blunt injuries (Klaas et al., 2002; ESCOP, 2003; Drogosz and Janecka, 2019). The therapeutic value of *A. montana* is attributed to secondary metabolites (Dall’Acqua et al., 2011; Kowalski et al., 2015; Sugier et al., 2017). Sesquiterpene lactones [SLs, esp. helenalin (H) and dihydrohelenalin (DH) esters with short chain carboxylic acids] are the main pharmacological active metabolites and phenolic acids and flavonoids contribute to the activity (Willuhn et al., 1983; Lange, 1998; Willuhn, 1998; Kos et al., 2005; Lin and Harnly, 2008; Merfort, 2010; Zidorn, 2010; Sülsen and Martino, 2018). The medicinally important flower heads are still partly obtained from collections on natural sites (e.g., Romania, Ukraine, France, and Spain; Kathe, 2006; Maurice et al., 2012).

Willuhn et al. (1994) suggested a minimum SL content of 0.4% of the dried flower head for standardization and distinguished two different chemotypes: (i) a central European type containing principally helenalin esters with a H/DH ratio higher than one and (ii) a Spanish type containing nearly exclusively dihydrohelenalin esters with a H/DH ratio lower than one (Willuhn et al., 1994).

The SL composition and content are known to be a function of multifold different abiotic and biotic factors controlling the biosynthesis and accumulation of these secondary metabolites (Verma and Shukla, 2015; Li et al., 2020). For example, it has been shown that the total SL content increases and the ratio of helenalin to dihydrohelenalin esters shifts from H to DH as *A. montana* plants mature (Schmidt et al., 1998; Douglas et al., 2004). Furthermore, the SL content can be influenced by genetic factors as well as abiotic site conditions, such as altitude, temperature and amount of rainfall (Spitaler et al., 2006; Perry et al., 2009; Seemann et al., 2010; Todorova et al., 2016). Up to now, soil related environmental conditions have not been investigated or revealed no significant correlation with the SL content. For example, Seemann et al. (2010) investigated in addition to rainfall, temperature and altitude the impact of soil pH, C/N ratio, potassium and phosphate, whereby the SL content remained unaffected by these soil parameters. However, soil is an important abiotic factor for the characterization of *A. montana* habitats. For illustration there is wide evidence that *A. montana* grows predominately on nutrient-poor and acidic soil (e.g., Ellenberg et al., 1991; Oberdorfer, 2001; Titze et al., 2020). Therefore, it can be assumed that *A. montana* tolerates nutrient poverty and acidic soil conditions. In contrast, *A. montana* seems to be less competitive in habitats with only slightly acidic soils (Dueck and Elderson, 1992; Pegtel, 1994; Hollmann et al., 2020). Hence, soil conditions greatly influence the vitality and occurrence of *A. montana* in natural environments by affecting plant species composition. This leads to the point that soil parameters such as pH, C/N, base saturation and cation exchange capacity might be potential drivers for enhanced secondary metabolite contents in *A. montana*.

Therefore, we consider varying small-scale site conditions to be of significant importance for the production of SL in *A. montana*. We focus on the impact of soil-related variables (pH, C/N ratio, base saturation, cation exchange capacity) and vegetation parameters (species richness, vegetation cover) on secondary metabolite contents in *A. montana*. Furthermore, we aimed at investigating whether these parameters would also affect the composition of SLs, thereby altering the chemotype of *A. montana*, bearing in mind that the chemotype change in wild plants in relation of habitat, vegetation, soil properties, as they showed some specific studies (Maksimović et al., 2007; Yeddes et al., 2018; Perrino et al., 2021). In order to test these assumptions, we took advantage of the fact that *A. montana* occurs not only on acidic soils above siliceous bed rock, but also on decalcified loamy soils above calcareous bed rock. Consequently, we investigated different *A. montana* populations naturally growing on soils from siliceous as well as calcareous geologies within the same geographical region and altitudinal belt, thereby covering a wide range of habitat conditions but controlling large scale climatic factors.

## Materials and Methods

### Study Sites and Design

Fieldwork was done in the Apuseni-Mountains in Romania on different localities in the municipality of Garda de Sus in the summer 2019. Two different geologies (siliceous or calcareous) with two management types each (mown or grazed grassland), were chosen to generate variability in biotic and abiotic conditions. Each geology was represented by nine grassland localities. The calcareous bedrock consisted of Wetterstein limestone from the Middle Triassic epoch of the Ladinian stage. The soil above the calcareous bedrock was characterized as loam. The siliceous bedrock consists of argillaceous shales from the lower Jurassic epoch of the Sinemurian, or green schist from the lower cretaceous. The soil above siliceous bedrock was oligotrophic to podzolic brown earth or ranker.

From a phytosociological point of view, two different types of grassland can be distinguished depending on the geology of the site. The grassland on calcareous sites can be described as a *Centaurea pseudophrygia*-Polygono-Trisetion society with a species-rich *Thymus pulegioides* formation. This plant community can be assigned to the association Polygono-Trisetion in the class Molinio-Arrhenatheretea (Brinkmann, 2006). The grassland on the siliceous sites can be described as a Festuca-rubra-Agrostis capillaris community. A clear assignment to a phytosociological class is difficult, as this society contains species of the Molinio-Arrhenatheretea class, the Festuco-Brometea class and the Nardo-Callunetea class (Ellenberg, 1952; Glavac, 1983; Reif et al., 2005). The investigated grassland sites of this study therefore include species of the Violo declinatae-Nardetum society, as well as the *Centaurea pseudophrygia*-Polygono-Trisetion society and sporadic some species of a semi-arid grassland society. The 10 species with the highest density per grassland type are listed together with *A. montana* in Supplementary Table 1.

Climatic variability among sites was taken into account by means of different factors. Thus, the study plots were placed close to each other in two areas whose centers are about 5 km apart. Within the two areas, the grassland sites are only a few 100 m apart and the maximum distance between two grassland sites is 10 km. All sites were located at a similar altitude between ca. 1,050 and 1,300 m a.s.l. and have a south-west to west orientation. In addition, the slope inclination was included as a measure of microclimatic differences.

The agricultural use of all sites is traditional and extensive: meadows with one annual cut for haymaking (four sites for each geology) and permanent pastures (five sites for each geology). The grazing regime was practiced at least for the last 7 years, but most likely for a much longer period. No pasture had ever received any additional mineral fertilizer. The annual grazing season is from May until August for at least two and up to 12 weeks by a maximum of eight cows and/or four horses per hectare. Once a year in July, the meadows were mown. Most of them received slight fertilization with manure annually or every second year. All meadows were additionally used for after grazing with a maximum of eight cows and two horses per hectare in September or October between 1 and 2 weeks.

To cover the variability within the grassland localities, we established 3–5 plots with varying *A. montana* density on every grassland site. In all investigated *A. montana* populations, three plots were established along a gradient from the center of the *A. montana* population with a very high *A. montana* density (P1) to a medium density (P3) to a very low density in the periphery of the *A. montana* population (P5). For large *A. montana* populations, in addition to the established three plots, two transitional density forms could be defined less rich (P2) and less poor (P4). Accordingly, we defined five *A. montana* density classes for every population from rich (P1), less rich (P2), middle (P3), less poor (P4) to poor (P5). Based on this the following average rosette numbers per plot where obtained for the relative density classes: rich = 764 rosettes/plot, less rich = 504 rosettes/plot, middle 300 rosettes/plot, less poor 145 rosettes/plot) to poor 99 rosettes/plot). Every plot had a size of 16 m^2^. To ensure plot homogeneity its shape was a 4 m × 4 m quadrat or a 2 m × 8 m rectangle. Within the nine siliceous grassland localities, we sampled 35 plots and 39 plots within the nine calcareous grassland localities, respectively. On every plot, the total number of vascular plant species plus their ground cover in percentage according to the vegetation scale after Londo extended by Zacharias (1996) were recorded. Samples for chemical analysis were collected and management and harvest date were recorded or assigned (see Supplementary Tables 2A,B for further information).

### *Arnica montana* Sampling

In June 2019, we randomly choose eight *A. montana* rosettes per plot and harvested one flower head at full blooming phase from each rosette. In the case of more than one flower head per rosette, we collected the uppermost flower head. The eight flower heads from each plot were pooled and dried at 45°C for around 38 h. Furthermore, we estimated the percentage of groundcover by *A. montana* rosettes on each plot according to the vegetation scale after Londo extended by Zacharias (1996).

### Quantification and Identification of Sesquiterpene Lactones

Sample preparation and quantification of the SL content in the harvested *A. montana* flower heads were done according to Willuhn and Leven (1991) and Flemming (2014), respectively, with α-santonin (Merck^®^, ≥ 99%) as an internal standard. In details, we homogenized and milled (MM400 Retsch^®^; *f* = 30.0 Hz; *t* = 30 s) the harvested and dried *Arnica* flower heads and used 250.0 mg of the pulverized plant material for the extraction. For the quantitative analysis of the SL content we used a GC 7702 A (Agilent Technologies^®^), equipped with a FID Varian 3900 for detection (detector temperature: 275°C; flow: 1 ml/min; injection volume: 1 μL; injector temperature: 250°C; split ratio: 20). The identification of the peaks was carried out under use of mass spectrometric measurements at the Faculty of Chemistry and Pharmacy of the University of Regensburg and literature values from Willuhn and Leven (1991). A master method assigned the retention times to the different SLs. We used the following formula to calculate the mass fraction of the different sesquiterpene lactones (MF_*SL*_ in mg g^–1^):


$${\text{MF}}_{\text{SL}}=\frac{{\text{A}}_{\text{SL}}*{\text{M}}_{\text{S}}*1000}{{\text{K}}_{\text{F}}*{\text{A}}_{\text{S}}*{\text{M}}_{\text{Arnica}}}$$

A_*S*_ = peak area santonin

A_*SL*_ = peak area SL

M_*S*_ = mass of santonin in [mg]

M_*Arnica*_ = dried plant material in [mg]

K_*F*_ = correction factors by Willuhn and Leven (1991)

We summed the individual fractions of all DH and H derivates to get the total SL content.

### Soil Measurements

On each plot, we collected four soil samples (depth 0–30 cm) using a Pürkhauer soil corer. The soil cores were extracted from each quarter of the plot, located in close proximity to *A. montana* plants. The soil samples were divided into topsoil (depth 0–10 cm) and subsoil (depth 10–30 cm), whereby only the topsoil samples were used for further investigations. We pooled the samples for each plot, dried them at 40°C for 24 h, and sieved them to remove particles above 2 mm. Subsequently, the pH was measured in distilled water, using a pH meter and an ion activity sensible glass electrode (Scott blue line CG 842). Afterward, the soil samples were milled and dried at 105°C for one night. Using a LECO CNS Elemental Analyzer (LECO Truspec™ CN; LECO Corporation, St. Joseph, MI, United States) the total carbon content (C) and nitrogen concentration (N) were determined, and the C/N ratio was calculated. To measure the effective cation exchange capacity, the soil samples were extracted with a 1 M NH_4_Cl solution. We determined the concentrations of each of the cations Al^3+^, Ca^2+^, Fe^3+^, K^+^, Mg^2+^, Mn^2+^ and Na^+^ with an inductively coupled plasma optical emission spectrometer (SPECTRO ICP-OES Ciros^CCD^ type). Afterward, the pH for every sample was measured again as described before. The cation concentrations including their loadings and the measured pH were used to calculate the effective cation exchange capacity (König, 2009). The base saturation was determined as percentage of cation exchange capacity occupied by the bases Na^+^, K^+^, Ca^2+^, Mg^2+^.

### Statistical Analyses

All statistical analyses were performed with R Studio 3.6.2 (R Core Team, 2020). The level of significance was set to *p* = 0.05. We used the following acronyms: *p* < 0.05 = *, *p* < 0.001 = ^**^, *p* <0.001 = ^***^. To evaluate the differences of the SL contents, soil variables, species richness and vegetation cover between factorial values we used boxplots. For assessing the significance levels between geologies and management types, we used a Wilcoxon test. To account for multiple testing *p*-values were adjusted according to Holm (1979). For the correlations between the environmental conditions and SL contents, we used a linear regression approach reporting *p*-values and adjusted *R*^2^. Finally, we performed a principal component analysis (PCA) to reveal the multivariate interactions between SL contents and soil as well as vegetation parameters. In order to show the correlations of the different variables with the principal dimensions we plotted a PCA correlation circle where the variables coordinates are illustrated by length and direction of respective arrows.

## Results

### Quantitative and Qualitative Analysis of *Arnica montana* Flower Heads

**FIGURE 1:**
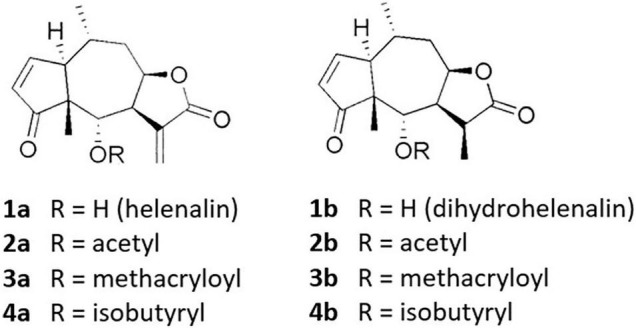
Sesquiterpene lactones of *Arnica montana* investigated in this study. Adopted from Douglas et al. (2004).

The quantitative analysis of *A. montana* flower heads from the Apuseni mountains (Romania) showed an average total SL content of 0.96 mg g^–1^, which represents the sum of the single contents of H, DH and three of their short chain esters each (Figure 1). Thereby, DH and its esters contributed around 74%, H and its esters around 26% to the total SL content. The main SL constituent was dihydrohelenalin acetate (Ac-DH) with an average content of 38%, followed by dihydrohelenalin methylacrylate (Met-DH) with around 27%. These two ingredients were the only SLs appearing in every plot sample. For a qualitative evaluation, we examined the relative contents of H and DH. The average H/DH, ratio, defined as the sum of all H derivatives to the sum of all DH derivatives was 0.39.

### Effects of Environmental Variability on the Sesquiterpene Lactones Content in *Arnica montana* Flower Heads

#### *Arnica montana* Density Classes

As one would expect, the percentage of ground cover by *A. montana* rosettes was significantly different between the five classes *A. montana* richness. The highest cover was observed on the *A. montana* rich plots and the lowest at the *A. montana* poor plots. The *A. montana* richness classes and the variability within the grassland localities showed no significant differences in the total SL content, the H/DH ratio or in the content of any of the single SLs. Furthermore, neither did the number of species nor the vegetation cover differ, nor showed the soil parameters any significant differences between the five *A. montana* richness classes.

#### Meadows vs. Pastures

The *A. montana* flower heads collected from meadows or pastures showed no significant differences in total SL content, H/DH ratio and contents of most of the single SL esters. Only the DH content of *A. montana* flower heads collected from meadows was significantly higher than from pastures (*p* < 0.05). According to the soil, environmental and vegetation parameters, no differences in altitude and vegetation cover were found between the two management classes. However, the vascular plant species number per plot was significantly higher on meadows (48 ± 2.42) than on pastures (38 ± 2.75; *p* < 0.01).

#### Geology: Calcareous vs. Siliceous Grassland Localities

The soil and vegetation parameters of the grassland communities differed considerably with respect to the two geologies (Table 1). The loamy soils on the calcareous bedrock showed significant higher pH values (5.19 ± 0.07), compared to the soils on the siliceous bedrock (4.49 ± 0.08; *p* < 0.001). In addition, the cation exchange capacity and the base saturation were significantly higher, whereas the C/N ratio was significantly lower on the calcareous grassland localities than on the siliceous grassland localities (*p* < 0.001). The number of species (*p* < 0.001) and the vegetation cover (*p* < 0.01) was significantly higher and the mean altitude (*p* < 0.001) was significantly lower at the calcareous sites compared to the siliceous sites. Whereby the marginal difference in altitude was presumably without any ecological effect (Table 1 altitude). No differences in slope inclination were found between the two geologies.

**TABLE 1:** Mean and standard error of the mean (std error) of various environmental parameters from *Arnica montana* grasslands in the Apuseni Mountains (Romania), distinguished between two geologies (s = siliceous, c = calcareous).

	Siliceous parent rock	Calcareous parent rock	s vs. c
	Mean ± std error	Mean ± std error	*p*-value
Soil pH	4.49 ± 0.14	5.19 ± 0.05	***
C/N ratio	15.51 ± 0.36	13.18 ± 0.14	***
cec	116.95 ± 4.99	200.63 ± 8.16	***
bs	43.14 ± 3.36	85.52 ± 2.34	***
Species no	33 ± 1.86	52 ± 1.2	***
Veg cover	81.89 ± 5.02	105.79 ± 5.55	**
Altitude	1,181 ± 13.8	1,111 ± 7.1	***

Only soil and environmental measurements differing significantly between the geology are shown: soil pH, C/N ratio, cec, cation exchange capacity; bs, soil base saturation; species no, vascular plant species number; veg cover, vegetation cover in percentage and altitude in m a.s.l.. P-values were calculated using a Wilcoxon test and level of significance is given (**p < 0.01; ***p < 0.001).

Furthermore, the total SL content and all single SL contents of the *A. montana* flower heads differed significantly between the geology of the grassland localities (Table 2). The flower heads of *A. montana* plants growing on grassland localities above calcareous parent rock showed a significant higher total SL content than the flower heads from *A. montana* plants growing above a siliceous parent rock (*p* < 0.001).

**TABLE 2:** Mean and standard error of the mean (std error) of different sesquiterpene lactone (SL) contents and the total SL content (mg g^–1^) in *A. montana* flower heads from Apuseni Mountains (Romania), distinguished between two geologies (s = siliceous, c = calcareous).

	Siliceous parent rock	Calcareous parent rock	s vs. c
	Mean ± std error	Mean ± std error	*p*-value
H	0.063 ± 0.009	0.124 ± 0.014	**
DH	0.082 ± 0.005	0.067 ± 0.006	*
Ac-H	0.056 ± 0.007	0.08 ± 0.007	*
Ac-DH	0.281 ± 0.011	0.429 ± 0.019	***
Ibut-H	0.02 ± 0.005	0.039 ± 0.005	*
Ibut-DH	0.025 ± 0.007	0.042 ± 0.007	*
Met-H	0.056 ± 0.007	0.095 ± 0.008	***
Met-DH	0.167 ± 0.006	0.276 ± 0.013	***
SL total content	0.75 ± 0.033	1.15 ± 0.046	***

*H, Helenalin; DH, dihydrohelenalin; Ac, acetyl; Ibut, isobutyryl; Met, methacryloyl esters.*

P-values were calculated using Wilcoxon test and level of significance is given (* p < 0.05; **p < 0.01; ***p < 0.001).

Additionally, the contents of most of the single SLs were significantly higher in flower heads of *A. montana* plants from calcareous grasslands compared to siliceous grasslands (*p* < 0.05). DH was the only ingredient exhibiting higher values on plots with siliceous soils (*p* < 0.05). Only the H/DH ratio showed no differences between the geology of the grassland localities.

#### Direct Correlations Between Environmental Factors and Sesquiterpene Lactone Content

**FIGURE 2:**
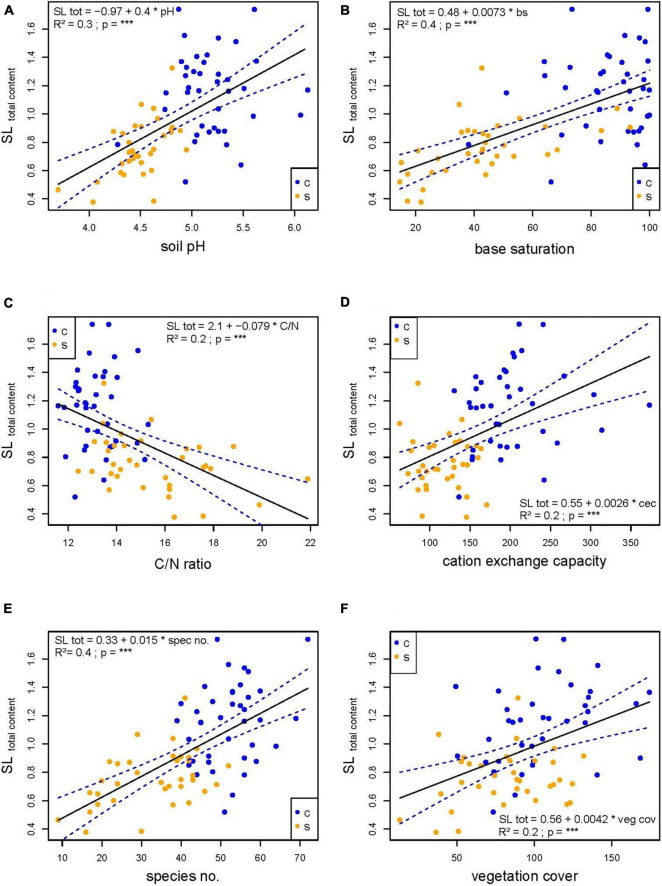
Linear models of different environmental parameters [soil pH **(A)**; base saturation **(B)**; soil C/N **(C)**; cation exchange capacity **(D)**; vascular plant species number **(E)**; vegetation cover **(F)**] and the total sesquiterpene lactone (SL) content (in mg g^–1^). The samples from grasslands localities with calcareous parent rock (c) are given in blue and the samples from grassland localities with siliceous parent rock (s) in orange. The regression equation, the r-squared (*R*^2^), the *p*-value, and the 95% confidence band are given for each relationship. Level of significance is given (*p < 0.05; **p < 0.01; ***p < 0.001).

In order to understand, which factors influenced SL contents, we examined the direct correlations between the environmental parameters and the SL content. We found significant correlations between soil parameters and the total SL content as well the H/DH ratio. The soil pH (*R*^2^ = 0.3, ^***^), base saturation (*R*^2^ = 0.4, ^***^) and cation exchange capacity (*R*^2^ = 0.2, ^***^) had a positive and soil C/N (*R*^2^ = 0.2, ^***^) a negative effect on the total SL content (Figures 2A–D). Furthermore, the species number (*R*^2^ = 0.4, ^***^, Figure 2E) and the vegetation cover (*R*^2^ = 0.2, ^***^, Figure 2F) had a positive and the altitude (*R*^2^ = 0.1, ^**^, data not shown) a negative effect on the total SL content. The H/DH ratio responded in the same way and was positively affected by soil pH, base saturation, species number, vegetation cover and negatively affected by the C/N ratio (Table 3). No significant relationship between the altitude and the H/DH ratio was found. Apart from this, most of the single SL contents and SL esters were influenced by the different soil parameters; just the DH content did not respond to any of the soil parameters and the slope inclination had no effect on the different ingredient contents (see Supplementary Table 3).

**TABLE 3:** Relationships between the H/DH ratio of the SL content in *A. montana* flower heads from Apuseni Mountains, Romania and environmental parameters: soil pH, base saturation (bs), C/N ratio, the species number per plot (species no.) and the vegetation cover in percentage (veg cover).

		*R* ^2^	*p*-value	corr
H/DH ratio	Soil pH	0.07	*	↗
	bs	0.09	**	↗
	C/N ratio	0.05	*	↘
	Species no.	0.09	**	↗
	Veg cover	0.06	*	↗

The r-squared (R^2^) and the p-values were calculated and level of significance is given (*p < 0.05; **p < 0.01; *** < 0.001).

#### Indirect Correlations Between Environmental Factors and Sesquiterpene Lactones Content

**FIGURE 3:**
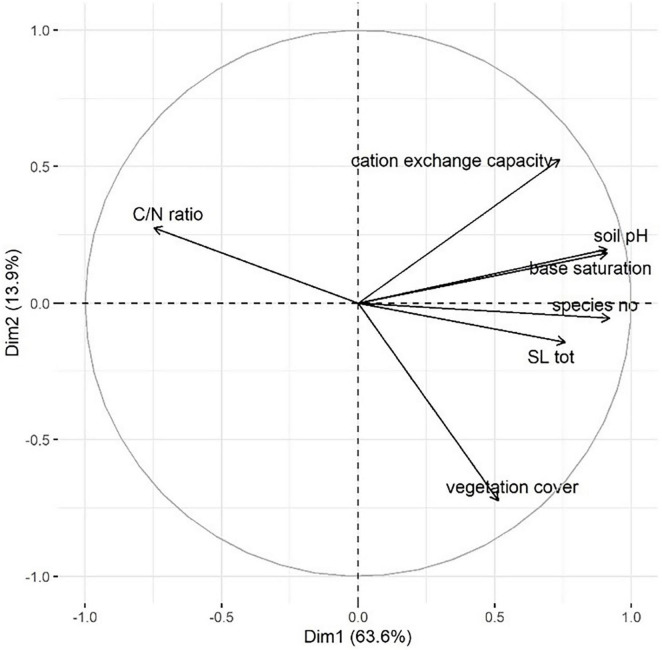
PCA correlation circle using *A montana* total SL contents and related environmental and vegetation parameters. PCA dimensions 1 and 2 are plotted. Correlations of the variables with the PCA dimensions are illustrated by the arrows; arrow direction is linked to the correlation with a certain PCA dimension, arrow length indicates strength of correlation.

Additionally, we plotted a PCA correlation circle using the total *A. montana* SL contents linked to environmental and vegetation parameters (Figure 3). The first two dimensions accounted for 63.6% (Dim1) and 13.9% (Dim2) to total inertia. The multivariate analysis revealed that soil pH, total SL content, base saturation, species number, cation exchange capacity and C/N ration were strongly correlated with PCA Dimension 1 (*r* > 0.7), whereas vegetation cover was correlated with Dimension 2 (*r* > 0.7). Furthermore, the analysis illustrated that soil pH, base saturation, species number and total SL content were closely positively interrelated.

## Discussion

This is the first study investigating the impact of different pedological parameters covering a relatively wide pH range on the production of secondary metabolites in *Arnica montana*.

We identify a clear relationship between soil conditions and sesquiterpene lactones in flower heads of *A. montana*. Since almost all investigated SLs responded to differing soil conditions this seems to be stable.

Using an *in situ* design for this study facing the complexity of a natural surrounding with varying ecological conditions, we could show that the growing conditions of *A. montana* are not restricted to a small pH range between 3.5 and 3.7 as reported by Seemann et al. (2010). Contrastingly, we found *A. montana* vigorously growing in a pH range from 3.7 to 6.2. These findings enabled us to study the relationships of SL contents with environmental conditions covering the widest soil pH range ever investigated in this context. Furthermore, we are the first to take into account base saturation and cation exchange capacity as possible drivers for the SL contents in *A. montana*, indicating soil nutritional status.

Our results appear to be generalizable, even though our total SL contents are lower than the European Pharmacopeia minimum of 4 mg/g SLs in *A. montana* flower heads (BfArM, 2017). Flemming (2014) investigated similar low SL contents in Romanian flower heads of *A. montana* with the same analytic method developed by Willuhn and Leven (1991) as we used. In terms of chemical composition, our results are similar to Willuhn et al. (1994) who identified the following main SL in *A. montana* flower heads: helenalin isobutyrate, helenalin 2-methylbutyrate, helenalin methylacrylate and helenalin tiglate. In addition, Seemann et al. (2010) revealed helenalin acetate as a fifth main compound.

We found only three of these five main SLs in our samples. The DH derivatives prevailed, specifically in form of dihydrohelenalin acetate and dihydrohelenalin methylacrylate. So far, dominating dihydrohelenalins with dihydrohelenalin methylacrylate as a main compound were found only in flower heads coming from Spain (the Spanish chemotype; Willuhn and Leven, 1991; Willuhn et al., 1994; Blaschek et al., 2007). Our results contrast with the investigations of Willuhn et al. (1994) and further studies who described *A. montana* flower heads from central European origins where helenalins dominated (Blaschek et al., 2007; Perry et al., 2009; Seemann et al., 2010). A possible explanation might be that the SL contents shift from H to DH with maturation of the plant, due to the dehydrogenase system (Schmidt et al., 1998). In accordance to Seemann et al. (2010), this dehydrogenase system works very rapid altering SL contents in response to plant phenology. To account for this circumstance, we chose flowers within the same maturation status by visual identification, defined in our study design. Although this method is considered an indicator with limited sensitivity for the exact determination of the flowering status, we were able to avoid a bias caused by a confounding effect of date of raw material collection (see Supplementary Figure 1).

In contrast to other studies, where no correlation between H/DH ratio and environmental factors was found (Willuhn et al., 1994; Seemann et al., 2010), we could show that H and DH react differently to the soil conditions and consequently, also the H/DH ratio was driven by the conditions of the soil. Therefore, we can show that not only the concentration but also the composition of the SLs responds to soil conditions. The *A. montana* plants analyzed in this study showed a chemical pattern that changed based on soil conditions. Plants growing on siliceous soils exhibited an H/DH ratio resembling the Spanish chemotype, whereas plants from calcareous were similar to the European type. These findings raise the question if the H/DH chemotypes are really a product of genetically fixed differences linked to populations from certain geographical regions or if environmental conditions, especially soil conditions of the geographical region modify the H/DH patterns.

In general, we found higher SL contents in *A. montana* flower heads growing in more suitable soil conditions i.e., moderate soil acidity and sufficient nutrient availability. Therefore, the relationship between *A. montana* secondary metabolites and soil seems to be caused by the soil chemical conditions. This suggests an influence of the soil nutritional status on the synthesis of SLs. Abiotic and biotic factors including minerals required by the plants for their growth and development also affect the biosynthesis of secondary metabolites in plants (Verma and Shukla, 2015). Hence, the concentration of secondary metabolites is likely to alter in a complex response to these multifunctional causes. The soil nutritional status is a crucial determinant for the biosynthesis and changes of plant secondary metabolites (Verma and Shukla, 2015). The results of our study provide empirical support for this framework and underline the importance of soil chemistry as a driving force for enhanced secondary metabolite contents in *A. montana.*

Another set of parameters that comes into play with SLs are the biotic and land-use conditions at a certain habitat (Verma and Shukla, 2015). Besides their pharmacological and therapeutic importance, most SLs function in a wide range of protective activities in plants (Padilla-Gonzalez et al., 2016). The main protective roles are as anti-herbivores (Burnett et al., 1974; Picman, 1986) and antimicrobials (Rodriguez et al., 1976) or as plant growth regulators, e.g., inhibiting the growth of competing plants (Kanchan and Jayachandra, 1980; Picman and Picman, 1984; Kelsey and Locken, 1987; Fischer et al., 1989; Fischer and Kathe, 2007). Some SLs are phytotoxic against certain species (McCahon et al., 1973; Fischer et al., 1989; Macías et al. 1996; 1999; 2000; Andolfi et al., 2013; Miranda et al., 2015).

Allelopathic interactions driven by secondary metabolites are likely to occur in a more stress-tolerant than competitive plant species including *A. montana*. Taking into account that the competing situation in the environment where *A. montana* occurs is affected by changes in the soil conditions, an increasing SL content with more suitable soil conditions can be assumed.

Plants growing on nutrient-poor grassland compete with one another for the low amount of available nutrients. The competition of roots for nutrients is a symmetric competition with a balanced relationships—every species is limited in their growth of above ground biomass due to the small nutrient availability (Schwinning and Fox, 1995; Thomas, 2018; Titze et al., 2020). With increasing nutrient supply, the competition tends to shift from below to above ground. When the roots compete less for the few nutrients, they tend to invest into the above ground biomass, and competition for lights tends to prevails. The competition for light is more asymmetrical where few fast growing species (often grasses) outcompete slow growing species like *A. montana* (Schwinning and Weiner, 1998; Thomas, 2018; Titze et al., 2020).

In our case, we assume the shift from soil/nutrient to light competition, also indicated by the increasing vegetation cover and a related increasing above ground biomass, which leads to an increasing light competition for *A. montana*. The higher SL content could be indicating that *A. montana* faces the increasing competition with an increasing investment into allelopathy against the other plant species. To test this new hypothesis further investigations are needed. In conclusion, this study highlights that SL contents are regulated by both abiotic and biotic environmental factors shedding light upon the complex plant-soil as well as plant-plant interactions in extensive grassland ecosystems.

## Data Availability Statement

The original contributions presented in the study are included in the article/Supplementary Material, further inquiries can be directed to the corresponding author/s.

## Author Contributions

AG, RL, and AR contributed to conception and design of the study. AG performed the sample collection in Romania and wrote the first draft of the manuscript. AG, MHa, and JH organized and performed the analyses of the ingredients. MHa wrote sections of the material and method part. MHe, JH, RL, and AR contributed to the design of specific experiments, drafted the work and revised it critically for important intellectual content. All authors contributed to manuscript revision, read, and approved the submitted version.

## Conflict of Interest

The authors declare that the research was conducted in the absence of any commercial or financial relationships that could be construed as a potential conflict of interest.

## Publisher’s Note

All claims expressed in this article are solely those of the authors and do not necessarily represent those of their affiliated organizations, or those of the publisher, the editors and the reviewers. Any product that may be evaluated in this article, or claim that may be made by its manufacturer, is not guaranteed or endorsed by the publisher.
