# Supplementary material for: Soil and Vegetation Drive Sesquiterpene Lactone Content and Profile in Arnica montana L. Flower Heads From Apuseni-Mountains, Romania
Source: Front Plant Sci. 2022 Jan 28;13:813939. doi: 10.3389/fpls.2022.813939 (PMC8832060; doi:10.3389/fpls.2022.813939)
Supplement: Supplementary file 1 [file Table_1.docx]

| **Supplementary Table 1:** | | **Plant species of the investigated grassland sites with highest density.** con = consitency as number of plots with occurance of this species; den = density as total ground cover by this plant species according to the vegetation scale after Londo extended by Zacharias (1996); n = total number of plots (39 on calcareous grasslands, 35 on siliceous grassland sites). | | | |
| --- | --- | --- | --- | --- | --- |
|  | **species name** | | **con** | **den** | **den/n** |
| **calcareous grassland sites** | *Arnica montana* L. | | 39 | 712.9 | 18.3 |
|  | *Agrostis capillaris* L. | | 26 | 260.7 | 6.7 |
|  | *Centaurea pseudophrygia* C. A. Mey. | | 35 | 242.7 | 6.2 |
|  | *Trollius europaeus ssp. transilvanicus* (Schur) Domin | | 27 | 227.5 | 5.8 |
|  | *Thymus pulegioides ssp. pulegioides* L. | | 30 | 198 | 5.1 |
|  | *Anthoxanthum odoratum* L. | | 39 | 176.1 | 4.5 |
|  | *Carlina acaulis ssp. acaulis* L. | | 28 | 165.3 | 4.2 |
|  | *Trifolium pratense* L. | | 35 | 158.8 | 4.1 |
|  | *Pimpinella major ssp. major* (L.) Huds. | | 39 | 155.7 | 4.0 |
|  | *Festuca rubra ssp. rubra* L. | | 39 | 153.7 | 3.9 |
|  | *Alchemilla vulgaris* L. | | 35 | 127.2 | 3.3 |
| **siliceous grassland sites** | *Vaccinium myrtillus* L. | | 24 | 562.7 | 16.1 |
|  | *Arnica montana* L. | | 35 | 484.9 | 13.9 |
|  | *Festuca rubra ssp. rubra* L. | | 33 | 250 | 7.1 |
|  | *Potentilla erecta* (L.) Raeusch. | | 35 | 237.4 | 6.8 |
|  | *Nardus stricta* L. | | 24 | 233 | 6.7 |
|  | *Vaccinium vitis-idaea* L. | | 18 | 181.5 | 5.2 |
|  | *Anthoxanthum odoratum* L. | | 30 | 145.7 | 4.2 |
|  | *Deschampsia flexuosa* (L.) Trin. | | 28 | 141.2 | 4.0 |
|  | *Agrostis capillaris* L. | | 29 | 113.4 | 3.2 |
|  | *Thymus pulegioides ssp. pulegioides* L. | | 28 | 89.1 | 2.5 |
|  | *Trifolium pratense* L. | | 22 | 84.5 | 2.4 |
