# Supplementary material for: Soil and Vegetation Drive Sesquiterpene Lactone Content and Profile in Arnica montana L. Flower Heads From Apuseni-Mountains, Romania
Source: Front Plant Sci. 2022 Jan 28;13:813939. doi: 10.3389/fpls.2022.813939 (PMC8832060; doi:10.3389/fpls.2022.813939)
Supplement: Supplementary file 2 [file Table_2.docx]

| **Supplementary Table 2A:** | | | | | | | | | | | | | | | | | | | | | | | | | | | | | | |
| --- | --- | --- | --- | --- | --- | --- | --- | --- | --- | --- | --- | --- | --- | --- | --- | --- | --- | --- | --- | --- | --- | --- | --- | --- | --- | --- | --- | --- | --- | --- |
| **Overview of all general, environmental and soil data of the investigated plots on calcareous parent rock**. loc = location of the grassland - located in the north (N) or the south (S) of Garda de Sus; geo = geology – c = calcareous parent rock, s = siliceous parent rock; m typ = management type – p = pasture, m = meadow; A. richness = A. montana richness class – 1(rich), 2(middle rich), 3(middle), 4(middle poor), 5(poor); size = plot size in meters; A. cover % = A. montana ground cover in %; veg cov = cover in percentage of all vascular plant species per plot; spec no = vascular plant species number per plot; altitude in m above sea level in m; slope inclination in °; cec = cation exchange capacity; bs = base saturation. | | | | | | | | | | | | | | | | | | | | | | | | | | | | | | |
| **plot name** | **loc** | **geo** | | **m typ** | | **A. richness** | | **size** | | **A. cover [%]** | | **veg cover [%]** | | **spec no** | | **altitude [m]** | | **slope**  **[°]** | | **soil pH** | | **C/N ratio** | | **cec** | | **bs** | | |  |  |
| KWe1N1 | N | C | | p | | 1 | | 2x8 | | 20 | | 133 | | 55 | | 1060 | | 18 | | 4.9 | | 12.4 | | 130.6 | | 64.2 | | |  |  |
| KWe1N2 | N | C | | p | | 2 | | 2x8 | | 20 | | 123.8 | | 55 | | 1060 | | 17 | | 5.2 | | 12.4 | | 192.7 | | 85.6 | | |  |  |
| KWe1N3 | N | C | | p | | 3 | | 2x8 | | 20 | | 135.1 | | 57 | | 1060 | | 12 | | 5.0 | | 12.3 | | 164.2 | | 72.8 | | |  |  |
| KWe1N4 | N | C | | p | | 4 | | 2x8 | | 12.5 | | 105.9 | | 50 | | 1060 | | 13 | | 5.0 | | 12.7 | | 149.4 | | 71.2 | | |  |  |
| KWe1N5 | N | C | | p | | 5 | | 2x8 | | 7.5 | | 98.8 | | 53 | | 1060 | | 14 | | 5.3 | | 12.5 | | 213.1 | | 92.4 | | |  |  |
| KWe2N1 | N | C | | p | | 1 | | 2x8 | | 12.5 | | 119.3 | | 49 | | 1100 | | 19 | | 4.9 | | 13.0 | | 212.2 | | 73.5 | | |  |  |
| KWe2N3 | N | C | | p | | 3 | | 2x8 | | 3.8 | | 82.5 | | 56 | | 1100 | | 21 | | 5.4 | | 13.2 | | 304.2 | | 98.1 | | |  |  |
| KWe2N5 | N | C | | p | | 5 | | 2x8 | | 1.8 | | 91.1 | | 60 | | 1100 | | 15 | | 6.1 | | 12.4 | | 373.3 | | 99.9 | | |  |  |
| KWe3N1 | N | C | | p | | 1 | | 2x8 | | 52.5 | | 136.3 | | 53 | | 1130 | | 7 | | 4.9 | | 13.5 | | 157.5 | | 64.0 | | |  |  |
| KWe3N3 | N | C | | p | | 3 | | 2x8 | | 4.2 | | 133 | | 45 | | 1130 | | 7 | | 4.8 | | 12.7 | | 158.1 | | 51.1 | | |  |  |
| KWe3N5 | N | C | | p | | 5 | | 2x8 | | 1.8 | | n.a. | | 48 | | 1130 | | 16 | | 5.0 | | 12.3 | | 196.8 | | 84.2 | | |  |  |
| KWe4N1 | N | C | | p | | 1 | | 2x8 | | 70 | | 83.6 | | 39 | | 1074 | | 13 | | 5.1 | | 11.6 | | 176.3 | | 81.9 | | |  |  |
| KWe4N2 | N | C | | p | | 2 | | 2x8 | | 60 | | 50.5 | | 47 | | 1074 | | 14 | | 5.1 | | 14.0 | | 175.8 | | 78.3 | | |  |  |
| KWe4N3 | N | C | | p | | 3 | | 2x8 | | 47.5 | | 99 | | 42 | | 1074 | | 13 | | 5.1 | | 12.7 | | 153.9 | | 70.0 | | |  |  |
| KWe4N4 | N | C | | p | | 4 | | 2x8 | | 30 | | 165.9 | | 40 | | 1074 | | 11 | | 5.1 | | 12.4 | | 157.9 | | 83.3 | | |  |  |
| KWe4N5 | N | C | | p | | 5 | | 2x8 | | 3.8 | | 140.7 | | 56 | | 1074 | | 7 | | 5.3 | | 13.6 | | 188.6 | | 95.8 | | |  |  |
| KWe5N1 | N | C | | p | | 1 | | 2x8 | | 20 | | 49.4 | | 46 | | 1173 | | 7 | | 5.1 | | 13.5 | | 194.9 | | 86.3 | | |  |  |
| KWe5N3 | N | C | | p | | 3 | | 2x8 | | 12.5 | | 168.6 | | 56 | | 1173 | | 11 | | 5.2 | | 14.9 | | 258.7 | | 97.2 | | |  |  |
| KWe5N5 | N | C | | p | | 5 | | 2x8 | | 4.2 | | 99.6 | | 59 | | 1173 | | 11 | | 5.3 | | 13.8 | | 209.1 | | 92.7 | | |  |  |
| KWi1N1 | N | C | | m | | 1 | | 2x8 | | 30 | | 134.7 | | 44 | | 1099 | | 7 | | 5.3 | | 13.8 | | 199.1 | | 91.1 | | |  |  |
| KWi1N2 | N | C | | m | | 2 | | 2x8 | | 20 | | 105.4 | | 52 | | 1099 | | 9 | | 5.3 | | 13.6 | | 182.9 | | 93.4 | | |  |  |
| KWi1N3 | N | C | | m | | 3 | | 2x8 | | 12.5 | | 121.1 | | 56 | | 1099 | | 12 | | 5.4 | | 13.1 | | 166.1 | | 93.2 | | |  |  |
| KWi1N4 | N | C | | m | | 4 | | 2x8 | | 7.5 | | 102.6 | | 56 | | 1099 | | 11 | | 5.3 | | 12.9 | | 203.6 | | 96.5 | | |  |  |
| KWi1N5 | N | C | | m | | 5 | | 2x8 | | 1.8 | | 115.9 | | 57 | | 1099 | | 7 | | 5.4 | | 13.9 | | 205.2 | | 98.5 | | |  |  |
| KWi2N1 | N | C | | m | | 1 | | 4x4 | | 40 | | 141.1 | | 52 | | 1115 | | 11 | | 4.9 | | 14.9 | | 214.5 | | 91.5 | | |  |  |
| KWi2N2 | N | C | | m | | 2 | | 4x4 | | 12.5 | | 174.5 | | 53 | | 1115 | | 12 | | 5.1 | | 14.0 | | 187.1 | | 90.5 | | |  |  |
| KWi2N3 | N | C | | m | | 3 | | 4x4 | | 7.5 | | 73.6 | | 51 | | 1115 | | 7 | | 4.9 | | 12.3 | | 136.5 | | 66.3 | | |  |  |
| KWi2N4 | N | C | | m | | 4 | | 4x4 | | 3.8 | | 74 | | 48 | | 1115 | | 4 | | 5.0 | | 11.9 | | 153.2 | | 83.3 | | |  |  |
| KWi2N5 | N | C | | m | | 5 | | 4x4 | | 2.2 | | 86.3 | | 45 | | 1115 | | 9 | | 5.0 | | 11.8 | | 151.1 | | 82.4 | | |  |  |
| KWi3N1 | N | C | | m | | 1 | | 2x8 | | 30 | | 101.1 | | 72 | | 1197 | | 10 | | 5.6 | | 13.7 | | 241.9 | | 99.4 | | |  |  |
| KWi3N2 | N | C | | m | | 2 | | 2x8 | | 20 | | 109.5 | | 69 | | 1197 | | 18 | | 5.5 | | 12.7 | | 227.9 | | 97.9 | | |  |  |
| KWi3N3 | N | C | | m | | 3 | | 2x8 | | 12.5 | | 92.1 | | 58 | | 1197 | | 14 | | 6.1 | | 12.8 | | 314.1 | | 100.0 | | |  |  |
| KWi3N4 | N | C | | m | | 4 | | 2x8 | | 3.8 | | 98.6 | | 64 | | 1197 | | 17 | | 5.6 | | 13.2 | | 241.0 | | 99.3 | | |  |  |
| KWi3N5 | N | C | | m | | 5 | | 2x8 | | 1.8 | | 77 | | 60 | | 1197 | | 13 | | 5.6 | | 13.1 | | 267.2 | | 99.5 | | |  |  |
| KWi4N1 | N | C | | m | | 1 | | 2x8 | | 47.5 | | 92.1 | | 44 | | 1082 | | 8 | | 4.3 | | 15.2 | | 153.1 | | 38.1 | | |  |  |
| KWi4N2 | N | C | | m | | 2 | | 2x8 | | 40 | | 70.8 | | 42 | | 1082 | | 10 | | 4.7 | | 15.3 | | 186.5 | | 82.9 | | |  |  |
| KWi4N3 | N | C | | m | | 3 | | 2x8 | | 12.5 | | 68.8 | | 47 | | 1082 | | 7 | | 5.2 | | 13.7 | | 196.2 | | 94.6 | | |  |  |
| KWi4N4 | N | C | | m | | 4 | | 2x8 | | 1.8 | | 87.7 | | 53 | | 1082 | | 8 | | 5.5 | | 13.5 | | 242.4 | | 98.5 | | |  |  |
| KWi4N5 | N | C | | m | | 5 | | 2x8 | | 0.6 | | 77.1 | | 43 | | 1082 | | 11 | | 5.3 | | 12.7 | | 187.8 | | 96.4 | | |  |  |
| **Supplementary Table 2B:** | | | | | | | | | | | | | | | | | | | | | | | | | | | |  |  |  |
| **Overview of all general, environmental and soil data of the investigated plots on siliceous parent rock**. loc = location of the grassland - located in the north (N) or the south (S) of Garda de Sus; geo = geology – c = calcareous parent rock, s = siliceous parent rock; m typ = management type – p = pasture, m = meadow; A. richness = A. montana richness class – 1(rich), 2(middle rich), 3(middle), 4(middle poor), 5(poor); size = plot size in meters; A. cover % = A. montana ground cover in %; veg cov = cover in percentage of all vascular plant species per plot; spec no = vascular plant species number per plot; altitude in m above sea level in m; slope inclination in °; cec = cation exchange capacity; bs = base saturation. | | | | | | | | | | | | | | | | | | | | | | | | | | | |  |  |  |
| **plot name** | **loc** | | **geo** | | **m typ** | | **A. richness** | | **size** | | **A. cover [%]** | | **veg cover [%]** | | **spec no** | | **altitude [m]** | | **slope**  **[°]** | | **soil pH** | | **C/N ratio** | | **cec** | | **bs** | | |  |
| SWe1S1 | S | | S | | P | | 1 | | 2x8 | | 30 | | 58.1 | | 24 | | 1095 | | 16 | | 4.3 | | 16.9 | | 164.0 | | 43.0 | | |  |
| SWe1S2 | S | | S | | P | | 2 | | 2x8 | | 12.5 | | 86.8 | | 29 | | 1095 | | 22 | | 4.4 | | 16.7 | | 160.0 | | 43.9 | | |  |
| SWe1S3 | S | | S | | P | | 3 | | 2x8 | | 1.8 | | 38.3 | | 29 | | 1095 | | 23 | | 4.5 | | 15.4 | | 140.3 | | 34.9 | | |  |
| SWe1S4 | S | | S | | P | | 4 | | 2x8 | | 1.8 | | 116.6 | | 43 | | 1095 | | 23 | | 5.0 | | 14.8 | | 146.9 | | 68.4 | | |  |
| SWe1S5 | S | | S | | P | | 5 | | 2x8 | | 1.8 | | 84.5 | | 39 | | 1095 | | 15 | | 4.8 | | 14.3 | | 105.5 | | 37.9 | | |  |
| SWe2S1 | S | | S | | P | | 1 | | 2x8 | | 30 | | 46.6 | | 17 | | 1170 | | 13 | | 4.1 | | 16.2 | | 146.3 | | 14.6 | | |  |
| SWe2S3 | S | | S | | P | | 3 | | 2x8 | | 20 | | 36.7 | | 16 | | 1170 | | 12 | | 4.0 | | 16.4 | | 145.9 | | 21.9 | | |  |
| SWe2S5 | S | | S | | P | | 5 | | 2x8 | | 3.8 | | 13.2 | | 9 | | 1170 | | 10 | | 3.7 | | 19.9 | | 171.1 | | 22.6 | | |  |
| SWe3N1 | N | | S | | P | | 1 | | 2x8 | | 20 | | 88.9 | | 36 | | 1241 | | 9 | | 4.4 | | 13.8 | | 73.2 | | 27.6 | | |  |
| SWe3N2 | N | | S | | P | | 2 | | 2x8 | | 12.5 | | 110.4 | | 37 | | 1241 | | 18 | | 4.4 | | 12.4 | | 86.5 | | 47.8 | | |  |
| SWe3N3 | N | | S | | P | | 3 | | 2x8 | | 4.2 | | 64.2 | | 40 | | 1241 | | 11 | | 4.5 | | 13.0 | | 96.4 | | 53.6 | | |  |
| SWe3N4 | N | | S | | P | | 4 | | 2x8 | | 2.2 | | 80.3 | | 38 | | 1241 | | 11 | | 4.3 | | 13.7 | | 85.6 | | 44.0 | | |  |
| SWe3N5 | N | | S | | P | | 5 | | 2x8 | | 1.8 | | 88.7 | | 38 | | 1241 | | 11 | | 4.6 | | 13.5 | | 62.9 | | 88.9 | | |  |
| SWe4S1 | S | | S | | P | | 1 | | 2x8 | | 47.5 | | 90.6 | | 20 | | 1110 | | 20 | | 4.4 | | 17.7 | | 91.7 | | 20.8 | | |  |
| SWe4S3 | S | | S | | P | | 3 | | 2x8 | | 30 | | 112.3 | | 27 | | 1110 | | 18 | | 4.8 | | 17.4 | | 99.4 | | 35.7 | | |  |
| SWe4S5 | S | | S | | P | | 5 | | 2x8 | | 12.5 | | 51.1 | | 30 | | 1110 | | 11 | | 4.6 | | 17.6 | | 90.8 | | 17.2 | | |  |
| SWe5S1 | S | | S | | P | | 1 | | 2x8 | | 30 | | 73.9 | | 17 | | 1116 | | 2 | | 4.5 | | 16.0 | | 90.7 | | 15.1 | | |  |
| SWe5S2 | S | | S | | P | | 2 | | 2x8 | | 20 | | 101 | | 23 | | 1116 | | 1 | | 4.6 | | 16.2 | | 95.6 | | 30.3 | | |  |
| SWe5S3 | S | | S | | P | | 3 | | 2x8 | | 20 | | 121.2 | | 19 | | 1116 | | 6 | | 4.5 | | 16.2 | | 96.1 | | 17.3 | | |  |
| SWe5S4 | S | | S | | P | | 4 | | 2x8 | | 7.5 | | 124.7 | | 24 | | 1116 | | 15 | | 4.6 | | 17.9 | | 105.6 | | 38.0 | | |  |
| SWe5S5 | S | | S | | P | | 5 | | 2x8 | | 1.8 | | 39.8 | | 19 | | 1116 | | 15 | | 4.3 | | 21.9 | | 122.2 | | 46.2 | | |  |
| SWi1S1 | S | | S | | m | | 1 | | 2x8 | | 20 | | 74.5 | | 43 | | 1078 | | 12 | | NA | | 14.5 | | 129.2 | | 50.2 | | |  |
| SWi1S3 | S | | S | | m | | 3 | | 2x8 | | 7.5 | | 113 | | 44 | | 1078 | | 12 | | 4.7 | | 14.2 | | 124.2 | | 55.5 | | |  |
| SWi1S5 | S | | S | | m | | 5 | | 2x8 | | 1.8 | | 89.5 | | 41 | | 1078 | | 18 | | 4.8 | | 13.5 | | 85.6 | | 42.7 | | |  |
| SWi2N1 | N | | S | | m | | 1 | | 2x8 | | 40 | | 53.2 | | 35 | | 1238 | | 8 | | 4.4 | | 17.6 | | 130.4 | | 42.7 | | |  |
| SWi2N2 | N | | S | | m | | 2 | | 2x8 | | 20 | | 112.5 | | 49 | | 1238 | | 8 | | 4.4 | | 14.2 | | 110.2 | | 65.2 | | |  |
| SWi2N3 | N | | S | | m | | 3 | | 2x8 | | 3.8 | | 65.7 | | 52 | | 1238 | | 10 | | 4.7 | | 13.7 | | 76.9 | | 44.8 | | |  |
| SWi2N4 | N | | S | | m | | 4 | | 2x8 | | 4.2 | | 82.2 | | 40 | | 1238 | | 11 | | 4.5 | | 13.1 | | 79.2 | | 64.4 | | |  |
| SWi2N5 | N | | S | | m | | 5 | | 2x8 | | 1.8 | | 54.6 | | 42 | | 1238 | | 12 | | 4.8 | | 13.6 | | 132.1 | | 93.3 | | |  |
| SWi3N1 | N | | S | | m | | 1 | | 4x4 | | 40 | | 52.9 | | 22 | | 1308 | | 14 | | 4.2 | | 18.8 | | 147.3 | | 40.2 | | |  |
| SWi3N3 | N | | S | | m | | 3 | | 4x4 | | 7.5 | | 88.8 | | 40 | | 1308 | | 10 | | 4.6 | | 13.9 | | 141.4 | | 43.0 | | |  |
| SWi3N5 | N | | S | | m | | 5 | | 4x4 | | 0.6 | | 100.5 | | 42 | | 1308 | | 10 | | 4.9 | | 13.9 | | 158.4 | | 83.5 | | |  |
| SWi4N1 | N | | S | | m | | 1 | | 4x4 | | 20 | | 123.3 | | 42 | | 1295 | | 10 | | 4.3 | | 15.1 | | 135.9 | | 25.6 | | |  |
| SWi4N3 | N | | S | | m | | 3 | | 4x4 | | 3.8 | | 95.6 | | 46 | | 1295 | | 13 | | 4.4 | | 14.0 | | 128.2 | | 41.1 | | |  |
| SWi4N5 | N | | S | | m | | 5 | | 4x4 | | 2.2 | | 132 | | 38 | | 1295 | | 11 | | 4.5 | | 15.0 | | 137.6 | | 48.1 | | |  |
|  |  | |  | |  | |  | |  | |  | |  | |  | |  | |  | |  | |  | |  | |  | | |  |
