# Supplementary material for: Soil and Vegetation Drive Sesquiterpene Lactone Content and Profile in Arnica montana L. Flower Heads From Apuseni-Mountains, Romania
Source: Front Plant Sci. 2022 Jan 28;13:813939. doi: 10.3389/fpls.2022.813939 (PMC8832060; doi:10.3389/fpls.2022.813939)
Supplement: Supplementary file 3 [file Table_3.docx]

| **Supplementary Table 3:** | | | | | | | | | | |
| --- | --- | --- | --- | --- | --- | --- | --- | --- | --- | --- |
| **Effects of soil, environmental and vegetation parameters on the different Sesquiterpene lactone contents.** H = helenalin; DH = dihydrohelenalin; Ac = acetyl; Met = metacryloyl; Ibut = isobutyryl; bs = base saturation; cec = cation exchange capacity; spec no = species number per plot; veg cover = vegetation cover in percentage; altitude in m above sea level; slope = slope inclination. All results calculated with R-studio, function lm() and summary. | | | | | | | | | | |
|  | **X** | **R²** | **p-value** | **corr** |  |  | **X** | **R²** | **p-value** | **corr** |
| **H** | **soil pH** | 0.20 | *** | **↗** |  | **DH** | **soil pH** |  | > 0.05 |  |
|  | **bs** | 0.21 | *** | **↗** |  |  | **bs** |  | > 0.05 |  |
|  | **cec** | 0.05 | * | **↗** |  |  | **cec** |  | > 0.05 |  |
|  | **C/N ratio** | 0.08 | ** | **↘** |  |  | **C/N ratio** |  | > 0.05 |  |
|  | **spec no**  **veg cover** | 0.22  0.18 | ***  *** | **↗**  **↗** |  |  | **spec no**  **veg cover** |  | > 0.05  > 0.05 |  |
|  | **altitude** |  | > 0.05 |  |  |  | **altitude** |  | > 0.05 |  |
|  | **slope** |  | > 0.05 |  |  |  | **slope** |  | > 0.05 |  |
| **Ac-H** | **soil pH** | 0.09 | ** | **↗** |  | **Ac-DH** | **soil pH** | 0.27 | *** | **↗** |
|  | **bs** | 0.14 | *** | **↗** |  |  | **bs** | 0.28 | *** | **↗** |
|  | **cec** |  | > 0.05 |  |  |  | **cec** | 0.23 | *** | **↗** |
|  | **C/N ratio** | 0.09 | ** | **↘** |  |  | **C/N ratio** | 0.21 | *** | **↘** |
|  | **spec no**  **veg cover** | 0.11  0.05 | **  * | **↗**  **↗** |  |  | **spec no**  **veg cover** | 0.33  0.07 | ***  * | **↗**  **↗** |
|  | **altitude** |  | > 0.05 |  |  |  | **altitude** | 0.09 | ** | **↘** |
|  | **slope** |  | > 0.05 |  |  |  | **slope** |  | > 0.05 |  |
| **Met-H** | **soil pH** | 0.13 | *** | **↗** |  | **Met-DH** | **soil pH** | 0.27 | *** | **↗** |
|  | **bs** | 0.13 | *** | **↗** |  |  | **bs** | 0.30 | *** | **↗** |
|  | **cec** | 0.05 | * | **↗** |  |  | **cec** | 0.28 | *** | **↗** |
|  | **C/N ratio** | 0.07 | * | **↘** |  |  | **C/N ratio** | 0.15 | *** | **↘** |
|  | **spec no**  **veg cover** | 0.12  0.11 | **  ** | **↗**  **↗** |  |  | **spec no**  **veg cover** | 0.26  0.05 | ***  * | **↗**  **↗** |
|  | **altitude** | 0.09 | *** | **↘** |  |  | **altitude** | 0.13 | *** | **↘** |
|  | **slope** |  | > 0.05 |  |  |  | **slope** |  | > 0.05 |  |
| **Ibut-H** | **soil pH** | 0.08 | ** | **↗** |  | **Ibut-DH** | **soil pH** |  | > 0.05 |  |
|  | **bs** | 0.14 | *** | **↗** |  |  | **bs** | 0.06 | * | **↗** |
|  | **cec** | 0.05 | * | **↗** |  |  | **cec** |  | > 0.05 |  |
|  | **C/N ratio** | 0.11 | ** | **↘** |  |  | **C/N ratio** | 0.05 | * | **↘** |
|  | **spec no**  **veg cover** | 0.11 | **  > 0.05 | **↗** |  |  | **spec no**  **veg cover** |  | > 0.05  > 0.05 |  |
|  | **altitude** |  | > 0.05 |  |  |  | **altitude** |  | > 0.05 |  |
|  | **slope** |  | > 0.05 |  |  |  | **slope** |  | > 0.05 |  |
| **SL total content** | **soil pH** | 0.33 | *** | **↗** |  | **H/DH ratio** | **soil pH** | 0.07 | * | **↗** |
|  | **bs** | 0.40 | *** | **↗** |  |  | **bs** | 0.09 | ** | **↗** |
|  | **cec** | 0.23 | *** | **↗** |  |  | **cec** |  | > 0.05 |  |
|  | **C/N ratio** | 0.23 | *** | **↘** |  |  | **C/N ratio** | 0.05 | * | **↘** |
|  | **spec no**  **veg cover** | 0.38  0.17 | ***  *** | **↗**  **↗** |  |  | **spec no**  **veg cover** | 0.09  0.06 | **  * | **↗**  **↗** |
|  | **altitude** | 0.08 | ** | **↘** |  |  | **altitude** |  | > 0.05 |  |
|  | **slope** |  | > 0.05 |  |  |  | **slope** |  | > 0.05 |  |
