# Supplementary figures and images for: Soil and Vegetation Drive Sesquiterpene Lactone Content and Profile in Arnica montana L. Flower Heads From Apuseni-Mountains, Romania
Source: Front Plant Sci. 2022 Jan 28;13:813939. doi: 10.3389/fpls.2022.813939 (PMC8832060; doi:10.3389/fpls.2022.813939)

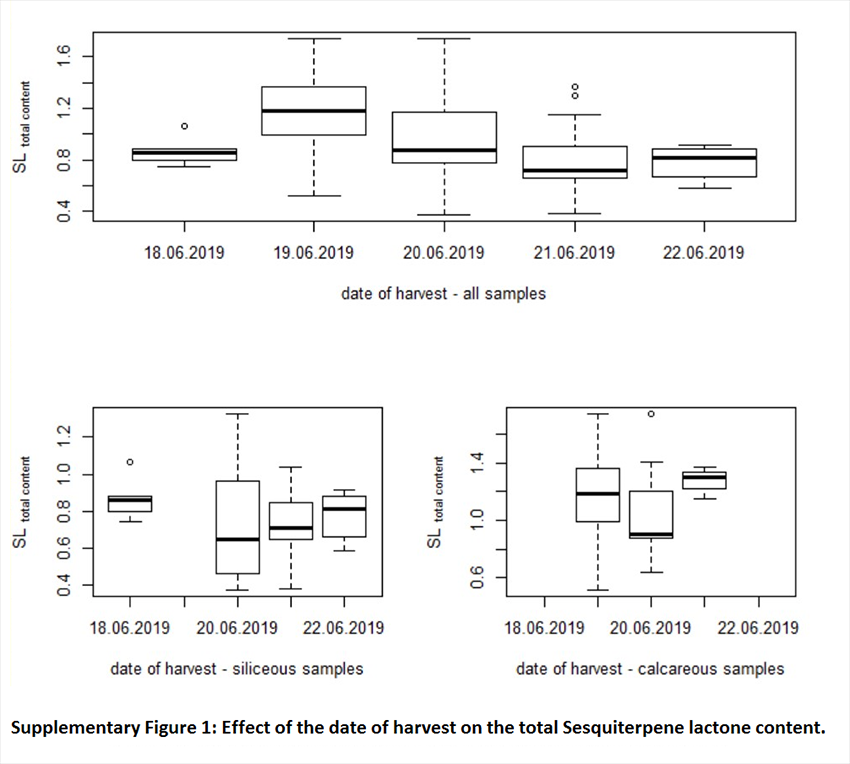

Supplement: Supplementary file 4 [file Image_1.tif]
